# A Post-segregational Killing Mechanism for Maintaining Plasmid PMF1 in Its *Myxococcus fulvus* Host

**DOI:** 10.3389/fcimb.2018.00274

**Published:** 2018-08-07

**Authors:** Ya-jie Li, Ya Liu, Zheng Zhang, Xiao-jing Chen, Ya Gong, Yue-zhong Li

**Affiliations:** State Key Laboratory of Microbial Technology, Institute of Microbial Technology, Shandong University, Qingdao, China

**Keywords:** pMF1 plasmid, nuclease, immune protein, co-transcriptional gene pair, post-segregational killing system, *Myxococcus fulvus*

## Abstract

Although plasmids provide additional functions for cellular adaptation to the environment, they also create a metabolic burden, which causes the host cells to be less competitive with their siblings. Low-copy-number plasmids have thus evolved several mechanisms for their long-term maintenance in host cells. pMF1, discovered in *Myxococcus fulvus* 124B02, is the only endogenous autonomously replicated plasmid yet found in myxobacteria. Here we report that a post-segregational killing system, encoded by a co-transcriptional gene pair of *pMF1.19* and *pMF1.20*, is involved in maintaining the pMF1 plasmid in its host cells. We demonstrate that the protein encoded by *pMF1.20* is a new kind of nuclease, which is able to cleave DNA *in vitro*. The nuclease activity can be neutralized by the protein encoded by *pMF1.19* through protein–protein interaction, suggesting that the protein is an immune protein for nuclease cleavage. We propose that the post-segregational killing mechanism of the nuclease toxin and immune protein pair encoded by *pMF1.20* and *pMF1.19* is helpful for the stable maintenance of pMF1 in *M. fulvus* cells.

## Introduction

Plasmids are self-replicating extra-chromosomal genetic elements that are dispensable to their bacterial host cells. They usually contain some essential genes for independent replication and segregation (Dmowski and Jagura-Burdzy, 2013), and some accessory genes (Hulter et al., 2017). The accessory genes may bring specific characters to their bacterial hosts, such as virulence factors in pathogens (Elwell and Shipley, 1980; Sengupta and Austin, 2011) and antibiotic resistance (Martinez and Baquero, 2002; Tett et al., 2007). However, the presence of plasmids also leads to extra metabolic burdens, which cause the host cells to be less competitive with their siblings. To decrease metabolic burden, plasmids are normally maintained at a low copy number, but this raises the risk of losing the plasmid during cell division. Plasmids have developed several maintenance mechanisms, such as sufficient copy numbers for daughter cells (Tabata et al., 1989; del Solar et al., 1998; Paulsson, 2002), site-specific resolution systems to avoid plasmid multimerization (Austin et al., 1981; Summers, 1998), partition systems (*par*) to actively segregate plasmid copies to daughter cells prior to cell division (Thomas, 2000; Ebersbach and Gerdes, 2005) and post-segregational killing (PSK) systems to kill competitive plasmid-free cells (Gerdes et al., 1986; Engelberg-Kulka and Glaser, 1999; Sengupta and Austin, 2011).

Myxobacteria are gram-negative bacteria that possess complex multicellular social behavior and large genomes (Shimkets, 1990; Dworkin, 1996; Han et al., 2013; Chen et al., 2016; Sharma et al., 2016). We previously isolated pMF1, which is the only endogenous plasmid yet found in myxobacteria, from *Myxococcus fulvus* 124B02 (Zhao et al., 2008). *M. xanthus–Escherichia coli* shuttle plasmids have been constructed based on the original replication region of pMF1 (Feng et al., 2012), and improved by containing the partitioning system of pMF1 (Sun et al., 2011); they are still losable from *M. xanthus* cells in the environment with no antibiotics selection pressure. In this study, we found a new nuclease and immune protein pair encoded by the co-transcriptional plasmid genes *pMF1.20* and *pMF1.19*. The presence of the gene pair made plasmids more stable in *Myxococcus* cells. We propose that together the two genes play a major role in the stable maintenance of plasmid pMF1 in *M. fulvus* cells using a post-segregational killing mechanism.

## Results

### *pMF1.19* and *pMF1.20* are co-transcribed, forming an operon in pMF1

**Figure 1:**
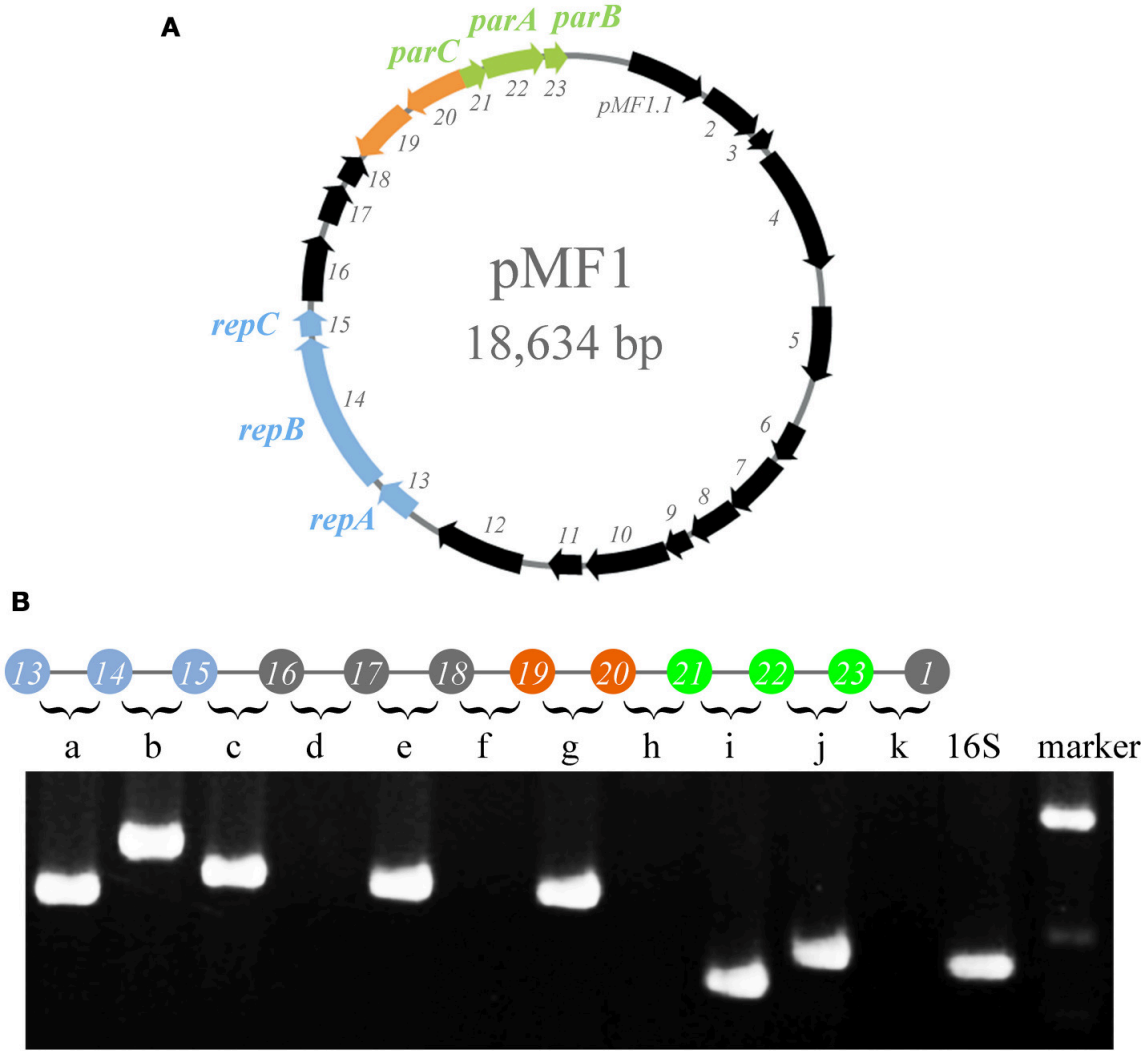
Operons in pMF1. **(A)** pMF1 consists of 23 genes. The blue arrows (*pMF1.13-pMF1.15*) represent the replication origin region. *pMF1.21-pMF1.23* (green arrows) is the partitioning system of pMF1. The orange parts are the two genes with an opposite direction to the others. **(B)** Co-transcription of the *pMF1.13-pMF1.23* genes by PCR mapping. The upper panel is a schematic drawing of the pMF1 genetic locus, and the lower panel is the amplification results of reverse transcription PCR for the fragments containing the 3′ end of the upstream gene, the intergenic sequence, and 5′ end of the downstream gene, corresponding to the sequence between two adjacent genes. The cDNA template was used to amplify the 16S rRNA gene as the positive control. The yield of RT-PCR product with the expected size indicated that the two neighboring genes were co-transcribed. The migration positions of molecular weight markers are indicated.

The genome sequence of pMF1 in *M. fulvus* 124B02 has been reported previously (Chen et al., 2016). The pMF1 plasmid is 18,634 bp in length and contains 23 predicted genes (*pMF1.1* to *pMF1.23*, Figure 1A and Table S1). We previously determined that genes *pMF1.13* to *pMF1.15* form the replication origin region and genes *pMF1.21* to *pMF1.23* constitute the partitioning system (Sun et al., 2011; Feng et al., 2012). BLASTp searching against GenBank databases showed that the predicted amino acid sequences of many genes in the pMF1 plasmid had homologs in various myxobacterial genome sequences (Table S1), all of which were functionally undetermined. The pieced construction of pMF1 with various myxobacteria-sourced genes suggests that the plasmid had been widely present in different myxobacteria, and had exchanged with the host genomes.

We performed transcriptomic analysis of the pMF1-harboring *M. fulvus* 124B02 to investigate the transcription of *pMF1.19* and *pMF1.20* genes (Figure S1). The results showed that the transcriptional level of *pMF1.20* was similar to that of the replication origin and partition system genes, while the transcriptional level of *pMF1.19* was much higher, indicating that *pMF1.20* and *pMF1.19* function in cells.

The transcriptomic results also indicated that *pMF1.19* and *pMF1.20* were in an operon, like *pMF1.13* to *pMF1.16* and *pMF1.21* to *pMF1.23*. The reverse transcriptional PCR result showed that the intergenic region between *pMF1.19* and *pMF1.20* could be amplified, while the intergenic regions between *pMF1.18* and *pMF1.19* or *pMF1.20* and *pMF1.21* were not (Figure 1B). Co-transcription in an operon suggest that the *pMF1.19* and *pMF1.20* genes are functionally related.

### The *pMF1.19*-*pMF1.20* gene pair have homologues in the 124B02 genome

Among the 23 genes, 21 were situated on the leading strand, while *pMF1.19* and *pMF1.20* were on the lagging strand. The most similar homologous genes of *pMF1.19* and *pMF1.20* were from *M. fulvus* 124B02 (Table S1). The amino acid sequence identities of pMF1.19 and pMF1.20 were 75 and 88% similar to MFUL124B02_RS18140 and MFUL124B02_RS18145, respectively. *MFUL124B02_RS18140* is downstream of *MFUL124B02_RS18145* in the genome, which is the same as the order of *pMF1.19* and *pMF1.20*. This result suggests that the *pMF1.19* and *pMF1.20* genes are probably repeats derived from the genome of *M. fulvus* 124B02, and *vice versa*.

Notably, the genome of *M. fulvus* 124B02 contains six homologs of *pMF1.20*, all of which are upstream of the homologs of *pMF1.19* (Table S2). All of these homologous gene pairs, apart from *MFUL124B02_RS18140* and *MFUL124B02_RS18145*, had low similarities with *pMF1.19* and *pMF1.20*. Interestingly, the phylogenies of the pMF1.19 homologs were completely consistent with those of their pMF1.20 homolog partners in the gene pairs (Figure S2). These results suggest that the *pMF1.19* and *pMF1.20* genes, as well as their homologous gene pairs in the genome of 124B02, co-evolved, and the functions of the paired genes are probably related but divergent.

### The *pMF1.19*-*pMF1.20* operon plays a role in plasmid maintenance in *myxococcus*

**Figure 2:**
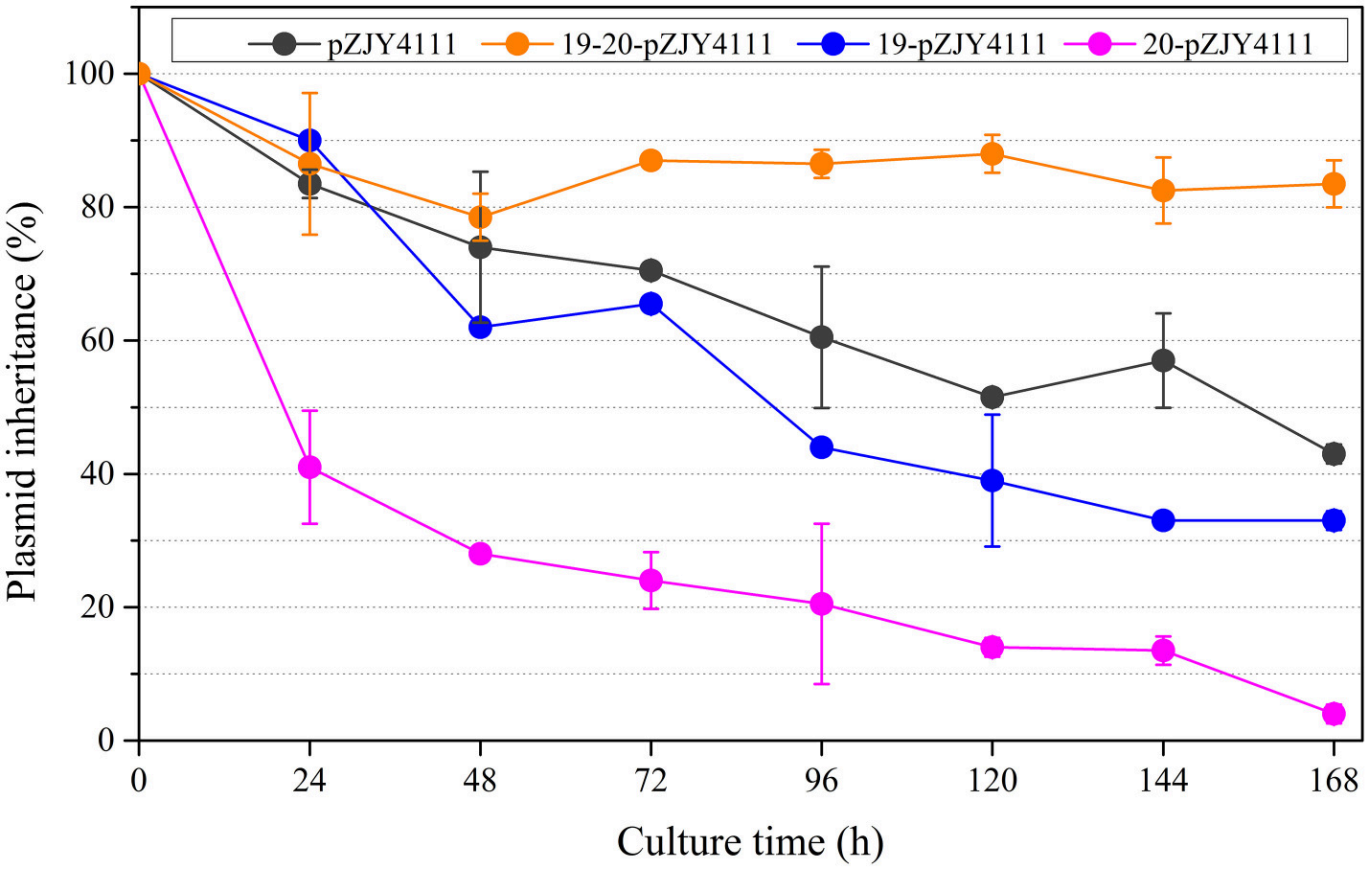
Effects of the presence of *pMF1.19* and/or *pMF1.20* on plasmid stabilities in *M. xanthus* DZ1. Plasmids 19-pZJY4111, 20-pZJY4111, and 19-20-pZJY4111 were constructed and electroporated into *M. xanthus* DZ1. The strains were cultivated in CTT medium without antibiotics for 168 h and plasmid retention was examined every 24 h.

The shuttle plasmid pZJY4111 contains the replication origin genes and partitioning genes of pMF1 (Sun et al., 2011). To investigate the functions of *pMF1.19* and/or *pMF1.20* in the plasmid, we introduced them into the pZJY4111 plasmid, producing plasmids 19-pZJY4111, 20-pZJY4111, and 19-20-pZJY4111 (the construction is shown in Figure S3). These recombinant plasmids were further separately transferred into the *M. xanthus* DZ1 strain, which grows dispersively in liquids (Breton et al., 1985; Zhao et al., 2008). We assayed the maintenance abilities of the plasmids in these strains, using the DZ1 transformant with plasmid pZJY4111 as a control. Without kanamycin selection, the retention of pZJY4111 in DZ1 was 43% after 168 h. A similar retention curve was observed in the 19-pZJY4111 plasmid stability test, but with weakly lower retention ability (Figure 2). However, after construction with the *pMF1.20* gene, the retention of 20-pZJY4111 plasmid was only 4% after 168 h, which suggests that *pMF1.20* alone was harmful to the hosting cells. In contrast, with the complete *pMF1.19*-*pMF1.20* operon, the retention of plasmid 19-20-pZJY4111 was 84% after 168 h, which was not only markedly higher than the retention of the 20-pZJY4111 plasmid, but also significantly higher than the retention of pZJY4111 or 19-pZJY4111 (*t*-test, *p* < 0.01). These results suggest that the co-presence of *pMF1.19* and *pMF1.20* had a stabilizing effect on the retention of the plasmid in *Myxococcus* cells. The *pMF1.19-pMF1.20* operon seemed to function using a post-segregational killing mechanism, i.e., plasmids with the operon could apparently eliminate plasmid-free cells, thus guaranteeing the survival of plasmid-containing cells in competition with plasmid-free cells. The *pMF1.20* gene might encode products that are toxic to the host cells whereas the product encoded by *pMF1.19* counteracts the toxicity of pMF1.20.

### The toxic effect of *pMF1.20* on cellular growth is suppressed by *pMF1.19*

**Figure 3:**
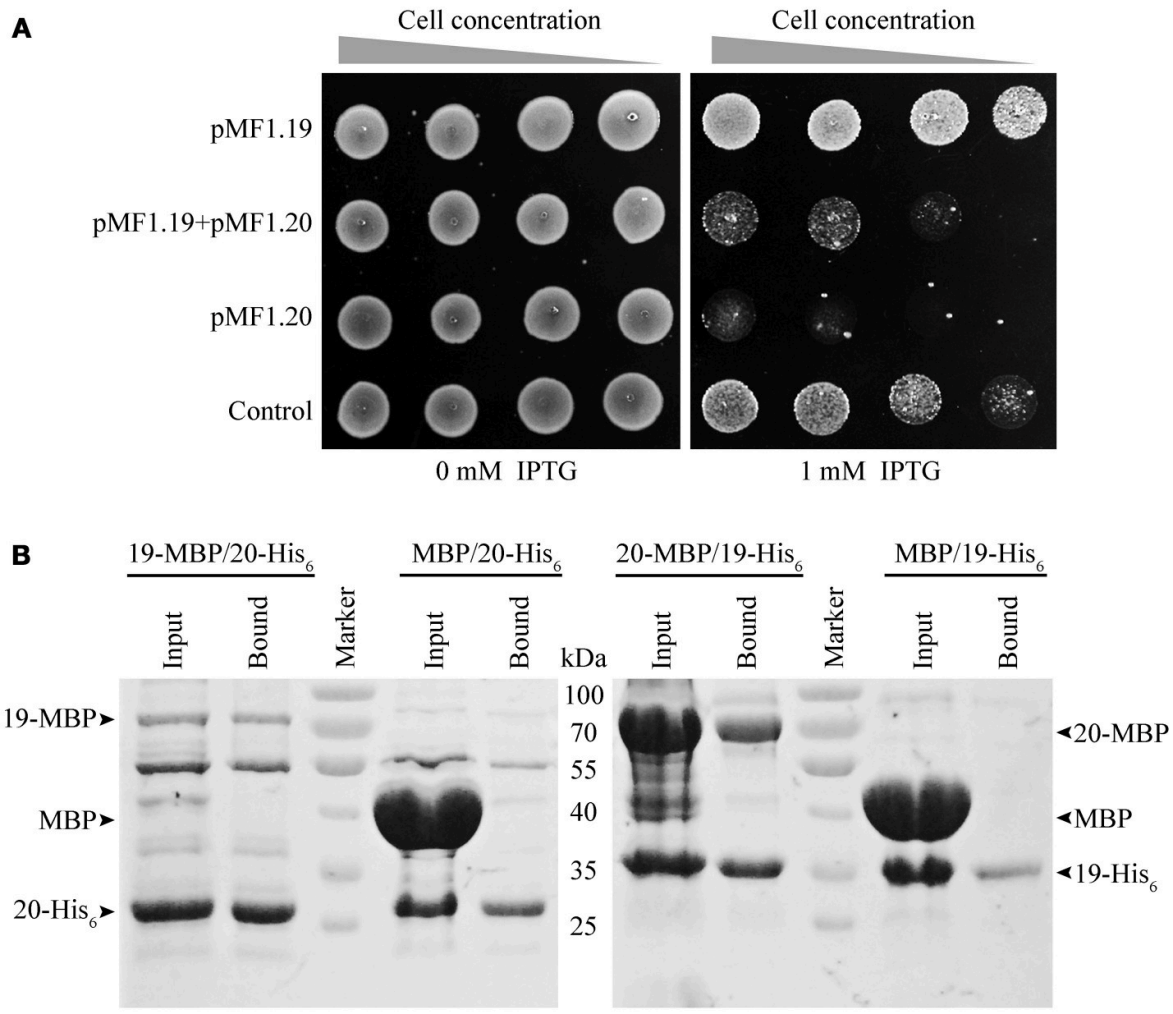
Results of *in vivo* and *in vitro* experiments with pMF1.19 and pMF1.20. **(A)** Growth abilities of the BL21 strains containing empty pMAL-c5x and pACYC-Duet plasmid (control), *pMF1.20*-harboring plasmid and empty pACYC-Duet, 19-pACYC and empty pMAL-c5x, and the two *pMF1.19*- and *pMF1.20*-harboring plasmids. The medium was with or without IPTG supplementation. Equal numbers of BL21 cells were five-fold serially diluted and spotted on the plates. Pictures were taken after overnight cultivation. **(B)** Pull-down experiments on the binding activities between pMF1.19 and pMF1.20. 19-MBP (71 kDa) and 20-MBP (69 kDa) were purified by amylose resin and 19-His_6_ (31 kDa) and 20-His_6_ (29 kDa) were purified by Ni^2+^ beads. 19-MBP (1.9 mg/ml, 300 μl) was incubated with 20-His_6_ (1.5 mg/ml, 300 μl). In a similar manner, 20-MBP (7 mg/ml, 150μl) was mixed with 19-His_6_ (1.5 mg/ml, 450 μl). MBP protein was incubated with 19-His_6_ or 20-His_6_ as negative controls. The input and bound samples were tested using SDS-PAGE. The PageRuler^TM^ Prestained Protein Ladder was used.

To determine the *in vivo* functions of *pMF1.19* and *pMF1.20*, we expressed them in *E. coli* cells. We performed a growth inhibition assay with *E. coli* strains (Figure 3A). The presence of plasmids had almost no effect on the growth of the *E. coli* strains. When 1 mM IPTG was added to the medium, the growth abilities of the strains with 19-pACYC/empty pMAL-c5x (pMF1.19) and with pACYC-Duet/pMAL-c5x (control) were weakened slightly. However, when the expression of *pMF1.20* was induced by IPTG, the growth ability of the strain containing 20-pMAL/pACYC-Duet (pMF1.20) was greatly decreased (Figure 3A). This result indicates that the presence of pMF1.20 is toxic to the cellular growth of *E. coli*. Compared with the single expression of *pMF1.20*, co-expression of *pMF1.20* and *pMF1.19* increased the growth of the strain. These results suggest that the *pMF1.20* expression product is toxic to cellular growth, and the *pMF1.19* expression product is capable of neutralizing the toxicity of pMF1.20.

We performed pull-down experiments to determine the *in vitro* binding interactions between the pMF1.20 and pMF1.19 proteins. 19-His_6_/20-MBP, 20-His_6_/19-MBP, 19-His_6_/MBP, and 20-His_6_/MBP mixtures were made. The mixtures were further extracted with Ni^2+^ beads, and the extracts were detected by SDS-PAGE to assay the binding abilities of the pMF1.20 and pMF1.19 proteins. If the two proteins were bound with each other, the 20-MBP and 19-MBP would be detectable in the extracts of 19-His_6_/20-MBP and 20-His_6_/19-MBP, respectively. As shown in the SDS-PAGE image (Figure 3B), when mixed with 19-His_6_ or 20-His_6_, the MBP protein did not appear in the extracts of the bound proteins. In contrast, 20-MBP and 19-MBP appeared in the extracts of the bound proteins when they were mixed with 19-His_6_ and 20-His_6_, respectively. These results demonstrate that pMF1.19 is able to bind to pMF1.20 *in vitro*.

### pMF1.20 possesses nuclease activity

**Figure 4:**
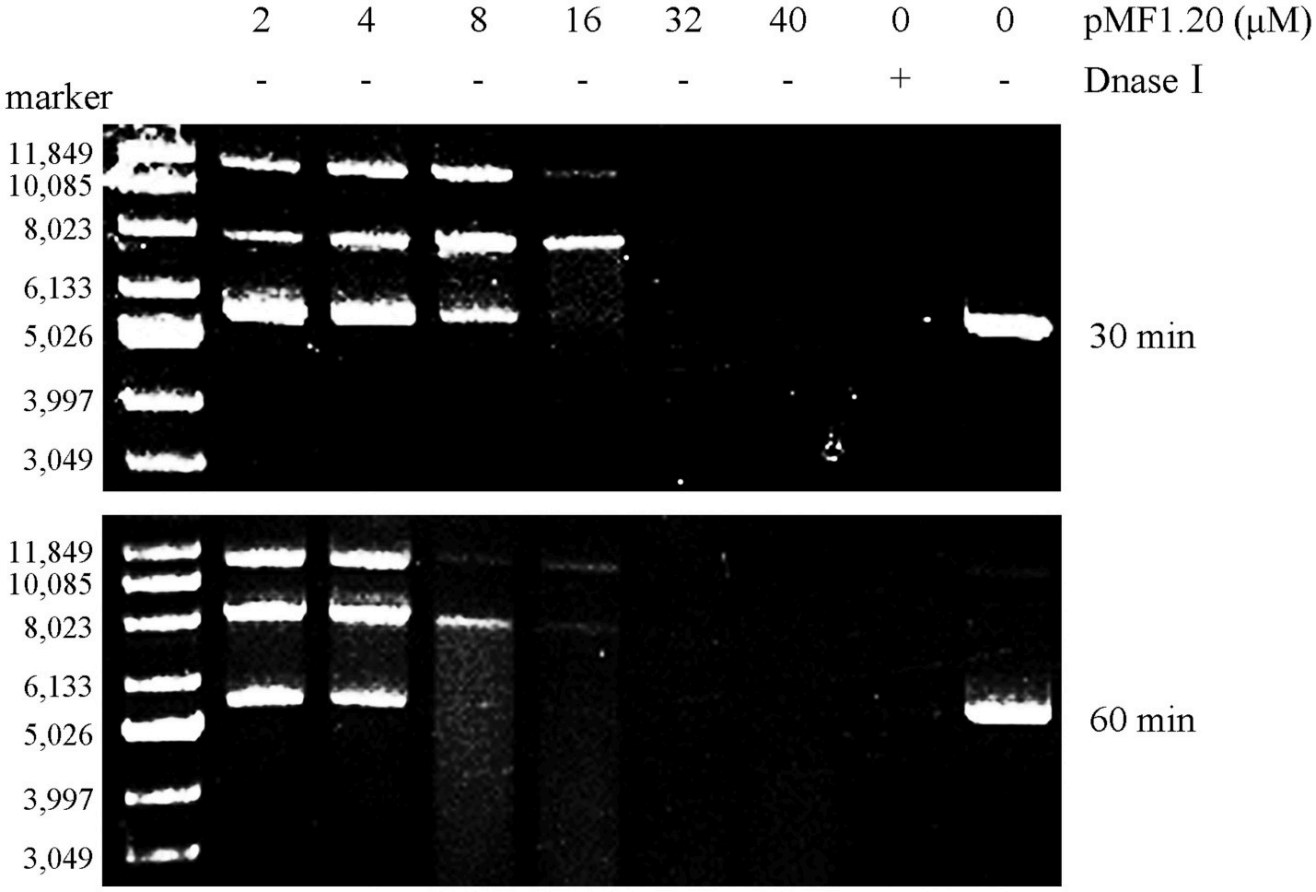
*In vitro* nuclease activity assay of the pMF1.20 proteins. Different concentrations of purified pMF1.20 were mixed with the supercoiled plasmid pMAL-c5x DNA for 30 and 60 min. DNase I was used for the positive control and the negative control contained no enzyme. The reactions were detected by gel and ethidium bromide staining.

Bioinformatics analysis revealed that the pMF1.20 protein and its homologs are all single-domain proteins containing a DUF2380 domain (pfam09533), and they were predicted to be lipoproteins with a type II signal peptide. The alignment of the amino acid sequences of pMF1.20 and its homologs indicated many conserved histidine sites (Figure S4). Some proteins with highly conserved histidine sites are known to be nucleases, in which histidine residues are in the active center (Midon et al., 2012; Belieres et al., 2015; Sivagnanam et al., 2016). We speculated that pMF1.20 was probably a new kind of nuclease. We therefore mixed purified pMF1.20 with supercoiled pMAL-c5x plasmid DNA in a nuclease reaction solution at 37°C for 30 or 60 min, followed by the DNA agarose gel electrophoresis. Dnase I was used as the control. At low concentrations, pMF1.20 loosened and linearized the plasmid DNA; at high concentrations, the protein digested the plasmid DNA completely (Figure 4). These results indicate that pMF1.20 possesses *in vitro* nuclease activity.

**Figure 5:**
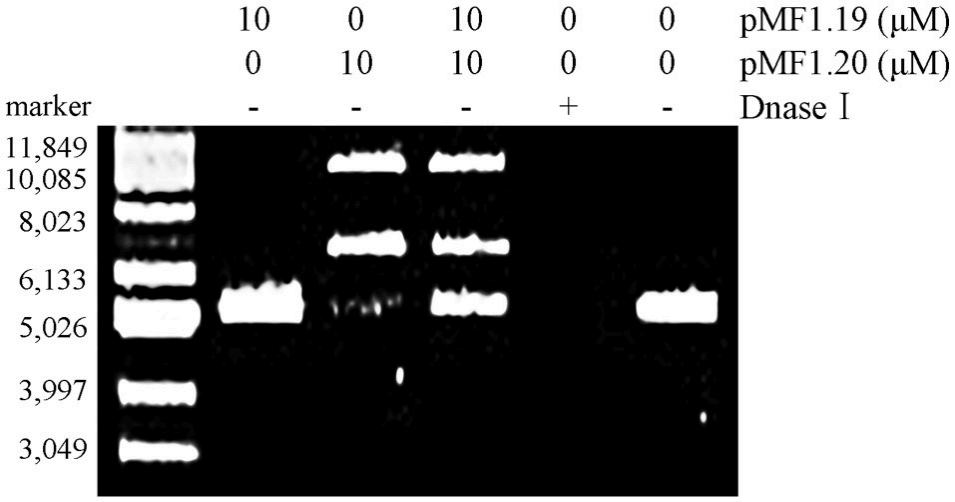
*In vitro* assay of pMF1.19 inhibition of pMF1.20 nuclease activity. Purified pMF1.19, pMF1.20 and equal parts pMF1.19 plus pMF1.20 were incubated with supercoiled pMAL-c5x DNA for 60 min. DNase I was used for the positive control and the negative control contained no enzyme.

We further mixed pMF1.20 with pMF1.19, and incubated the mixture with supercoiled pMAL-c5x plasmid DNA in a nuclease reaction solution at 37°C for 60 min. The results showed that the presence of 10 μM pMF1.19 alone did not lead to loosening, linearization, or digestion of the supercoiled DNA, whereas incubation with 10 μM pMF1.20 for 60 min caused the supercoiled DNA to disappear (Figure 5). Comparatively, the co-presence of 10 μM pMF1.19 and 10 μM pMF1.20 slowed the activities of pMF1.20 on the DNA substrate. These results suggest that the binding of pMF1.20 with pMF1.19 inhibited the enzymatic activity of the nuclease.

## Discussion

Lowering the copy number is a strategy of plasmids to reduce the metabolic burden on their cell host. However, a low copy number means a high risk of loss, and some mechanisms for retaining plasmids are necessary. Although the gene sequence and the replication origin region and partitioning system of pMF1 had been determined previously (Zhao et al., 2008; Sun et al., 2011; Feng et al., 2012), the known mechanisms were not sufficient to explain the long-term maintenance of pMF1 in *M. fulvus*. In this study, we revealed that the presence of the *pMF1.19*-*pMF1.20* operon can improve plasmid maintenance. We determined that *pMF1.20* encoded a novel nuclease protein, which was able to bind with pMF1.19 proteins. The *in vivo* and *in vitro* experiments showed that binding with pMF1.19 repressed the enzymatic activities of the pMF1.20 proteins. Notably, in *M. fulvus* 124B02, the expression of *pMF1.19* was approximately eight-fold that of *pMF1.20*, which implies that neutralizing the toxicity of the pMF1.20 nuclease required high concentrations of pMF1.19 proteins. We propose that the *pMF1.19*-*pMF1.20* operon probably functions like a PSK system for the maintenance of plasmids in *M. fulvus* cells.

The PSK system has been classified into two groups according to the type of the antidote. The antidote for the type I system is an antisense RNA complementary to the mRNA of the toxin gene, such as *hok-sok* from plasmid R1 (Gerdes et al., 1997). In the type II system, the antidote, as well as the toxin, is protein. In plasmid carrying cells, the two proteins form an inactive complex, which prevent the toxin from killing the cell. Plasmids F (*ccdA-ccdB*), P1 (*phd-doc*), and R1 (*kid-kis*) are well-understood type II PSK systems (Hayes and Van Melderen, 2011). For example, CcdB from the F plasmid and ParE from the RK2 plasmid influence the DNA gyrase actions (Jiang et al., 2002; Van Melderen, 2002). The Kid toxin of plasmid R1 is an RNase cleaving at the 5′-UUACU-3′ site to inhibit the expression of host gene so that stops cell growth, which is inactivated after binding with the Kis protein (Pimentel et al., 2005; de La Cueva-Mendez and Pimentel, 2007). The pMF1.20 toxin from pMF1 acts as nuclease effector, which may be delivered into neighboring cells and kill the co-existing plasmid-free cells that lack the corresponding immune proteins.

pMF1 is the only endogenous autonomously replicated plasmid that has been found in myxobacteria. The replication and partitioning systems are essential for the stable maintenance of pMF1 in *Myxococcus* cells. The nuclease-effector and immunity system consisting of *pMF1.20-pMF1.19* is also significant for the stable maintenance of pMF1. pMF1.20 has a lipoprotein signal peptide at the N-terminus of the protein, implying that the protein is probably located in the cellular membrane. Vassallo et al. reported that in the outer-membrane exchange system of *Myxococcus*, the TraA proteins are able to transfer nuclease proteins to adjacent cells and kill cells with no immune proteins (Vassallo et al., 2017). We speculate that pMF1.20 might be secreted to the target cells by a system similar to outer-membrane exchange. Further evidence of such a system is needed.

In the pMF1 plasmid, the direction of *pMF1*.*20* and *pMF1.19* is different from the other pMF1 genes. In addition, the most similar homologs of *pMF1.20* and *pMF1.19* are from *M. fulvus* 124B02, while the most similar homologs of other genes in pMF1 are not. There are at least six pairs of homologs of *pMF1.20-pMF1.19* in *M. fulvus 1*24B02. Thus, we speculate that the *pMF1.20-pMF1.19* operon is the latest member of pMF1 and is derived from the genome of *M. fulvus* 124B02. The new member *pMF1.20-pMF1.19* constitutes a post-segregational killing system of pMF1. The stable maintenance of pMF1 relies on cooperation between the replication, partitioning and post-segregational killing systems in *M. fulvus* 124B02.

## Experimental procedures

### Plasmids, strains, and culture conditions

The plasmids and bacteria used in this study are listed in Table S3. The *M. xanthus* strains were cultivated at 30°C in casitone-based rich-nutrient CTT agar or CTT broth (Hodgkin and Kaiser, 1977). *E. coli* cells were cultivated at 37°C on Luria-Bertani (LB) agar or in LB broth. When required, final concentrations of 40 μg/ml kanamycin, 100 μg/ml ampicillin, 34 μg/ml chloramphenicol were added to the medium.

### RNA extraction and reverse transcriptional PCR

Total RNA of pMF1-harboring *M. fulvus* 124B02 was extracted using an SV Total RNA Purification Kit (Promega USA). Residual genomic DNA (gDNA) was removed and cDNA was synthesized using a Prime Script^TM^ RT Reagent Kit with gDNA Eraser (Takara, China).The 16S rRNA gene sequence was amplified from the cDNA template to evaluate the quality of cDNA sample. The cDNA products were further amplified using specific primer pairs to determinate the co-transcription.

### Construction of plasmids for introduction into *myxococcus*

The *pMF1.19, pMF1.20*, and *pMF.19-pMF1.20* genes were cloned from pMF1, digested with BamH I and Kpn I and ligated into *M. xanthus-E. coli* shuttle plasmid pZJY4111 (which was also digested with BamH I and Kpn I) to obtain 19-pZJY4111, 20-pZJY4111, and 19-20-pZJY4111. The primers used in this study are listed in Table S4. The recombinant plasmids were electroporated into *M. xanthus* DZ1 according to the protocol described previously (Kashefi and Hartzell, 1995).

### Plasmid stability assay

To test stability, *M. xanthus* DZ1 strains harboring the plasmids were grown to the late exponential phase in liquid CTT medium supplemented with 40 μg/ml kanamycin. Then we diluted the cultures by 1:25 in fresh CTT liquid medium with no antibiotics and grew them at 30°C and 200 rpm. After 24 h of incubation, the cultures were serially diluted and plated on CTT agar without antibiotics. The dilutions and plating were routinely repeated every 24 h until 168 h of incubation. In each round, 100 single colonies were patched onto CTT agar with and without kanamycin, and plasmid stability was measured as the percentage of antibiotic-resistant clones (Sun et al., 2011; Shen et al., 2016). The data presented are the averages of three independent experiments.

### Pull-down assay for protein–protein interaction

We inserted *pMF1.19* in pACYC-Duet and pMAL-c5x, following the His_6_ and MBP fragments, respectively, to produce His_6_-tagged pMF1.19 (19-His_6_) and MBP-tagged pMF1.19 (19-MBP). The *pMF1.20* gene was similarly inserted into pET28a and pMAL-c5X to produce His_6_-tagged pMF1.20 (20-His_6_) and MBP-tagged pMF1.20 (20-MBP), respectively. We purified 19-His_6_ and 20-His_6_ using Ni beads, separately. 19-MBP and 20-MBP were purified by amylose resin, separately. The 19-His_6_ and 20-MBP mixture was incubated with Ni beads at 4°C for 2 h. We also incubated the MBP and 19-His_6_ mixture with Ni beads as the control. In turn the 19-MBP and 20-His_6_ mixture was incubated with Ni beads at 4°C for 2 h and MBP with 20-His_6_ as the control. After incubation, the proteins extracted by the beads were tested by SDS-PAGE.s.

### Protein expression and purification

The proteins were expressed in *E. coli* BL21 (DE3), induced by the addition of 0.1 mM of IPTG when the OD_600_ value of the culture reached 1.0. The BL21 cells were grown at 37°C in LB broth with antibiotics. After the addition of IPTG, the cultures were grown at 16°C for 20 h. The cells were then collected and resuspended in lysis buffer (25 mM Tris-HCl, pH 8.0, 200 mM NaCl and 5% glycerol, pH 8.0) and lysed via ultrasonication. The mixtures were centrifuged at 4°C for 30 min at 12,000 rpm. The soluble proteins were mixed with Ni beads (GE Healthcare, Sweden) or amylose resin (New England Biolabs) according to the manufacturer's protocols.

### Nuclease cleavage assay

19-MBP was purified using amylose resin (New England Biolabs) and 20-His_6_ was purified using Ni beads (GE Healthcare, Sweden). The substrate was supercoiled pMAL-c5x plasmid DNA, which was extracted using a TIANprep Mini Plamid Kit (Tiangen, China). Ten micrometers of purified pMF1.20 protein and 0.5 μg DNA were incubated for 30 and 60 min at 37°C in a reaction buffer (20 mM Tris-HCl, 200 mM NaCl and 5% glycerol, pH 8.0). The reaction was stopped by the addition of phenol/chloroform/isoamyl-alcohol, and the solutions were examined using 0.8% agarose gel electrophoresis. A supercoiled DNA ladder marker (Takara, China) was used.

### Growth inhibition assay in *E. coli*

*pMF1.19* was inserted into pACYC-Duet (19-pACYC), and the promoter was replaced with a constructive promoter. *pMF1.20* was inserted in pMAL-c5x (20-pMAL) following an IPTG-inducible promoter. The two compatible plasmids were introduced into *E. coli* BL21 simultaneously. In a similar manner, 20-pMAL with empty pACYC-Duet, 19-pACYC with empty pMAL-c5x and pACYC-Duet with pMAL-c5x were also separately introduced into the BL21 strain simultaneously as the control. Overnight LB cultures of the *E. coli* BL21 strains were five-fold serially diluted and spotted onto the LB agar with and without 1 mM IPTG. Photographs were taken after overnight incubation.

### Bioinformatics analyses

The gene information of plasmid pMF1 was obtained from the NCBI genome database (NC_010372.1). Using the amino acid sequences of pMF1.19 and pMF1.20 as query sequences, we searched for homologs in the *M. fulvus* 124B02 genome with BLASTp (Boratyn et al., 2012). Multiple sequence alignments of the full-length protein sequences of the pMF1.19 homologs and pMF1.20 homologs were implemented using MAFFT (Katoh and Standley, 2013). Phylogenetic analysis was conducted using the bootstrap neighbor joining method in MEGA version 7 (Kumar et al., 2016). A sequence logo was generated as a graphical representation of multiple sequence alignment (Crooks et al., 2004). The conserved domain information was obtained from the CDD protein family (Marchler-Bauer et al., 2017).

## Author contributions

YJL and YZL designed researches. YJL, YL, XC, ZZ, and YG performed researches. YJL, YL, XC, ZZ, and YG analyzed data. YJL and YZL wrote the paper. YZL provided funding for the project.

### Conflict of interest statement

The authors declare that the research was conducted in the absence of any commercial or financial relationships that could be construed as a potential conflict of interest.

## Supplementary material

Figure S1 Transcriptional analyses of the *pMF1.13-pMF1.23* genes in pMF1. **(A)** The transcriptional levels revealed by transcriptomic analysis. **(B)** The transcriptional levels of *pMF1.19* and *pMF1.20* by qPCR.

Figure S2 Comparison of the phylogenies of the amino acid sequences of pMF1.19 and pMF1.20 and their homologues in *Myxococcus fulvus* 124B02.

Figure S3 Construction of the 19-pZJY4111, 20-pZJY4111 and 19-20-pZJY4111 plasmids.

Figure S4 Consensus sequence of pMF1.20 and its homologues in Myxococcus fulvus 124B02. The sequence diversity at each position was assessed by multiple sequence alignment and displayed as a WebLogo.

Table S1 Protein Details for pMF1.

Table S2 Homologues in Myxococcus fulvus 124B02 of pMF1.19 and pMF1.20.

Table S3 Strains and plasmid used in this study.

Table S4 Primers used in this study.
