# Supplementary figures and images for: A Post-segregational Killing Mechanism for Maintaining Plasmid PMF1 in Its Myxococcus fulvus Host
Source: Front Cell Infect Microbiol. 2018 Aug 7;8:274. doi: 10.3389/fcimb.2018.00274 (PMC6091211; doi:10.3389/fcimb.2018.00274)

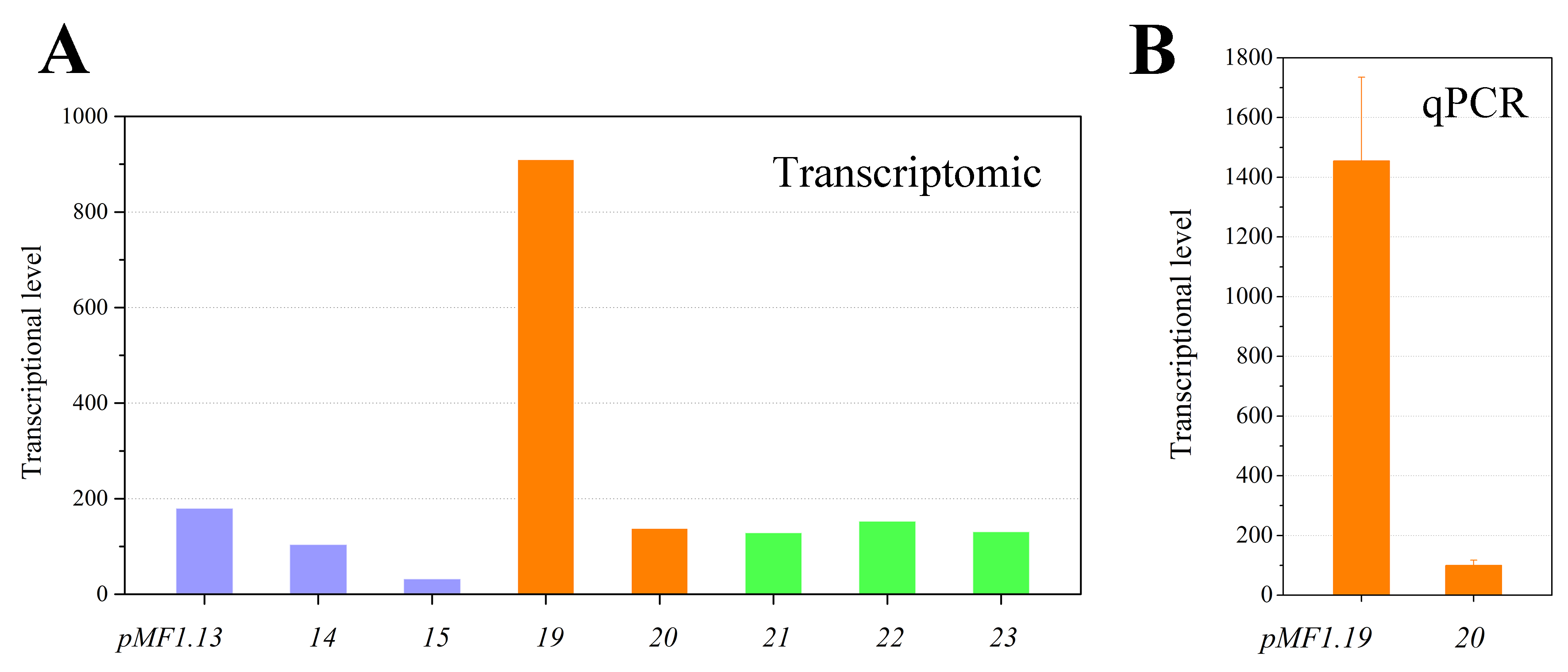

Supplement: Figure S1 — Transcriptional analyses of the pMF1.13-pMF1.23 genes in pMF1. (A) The transcriptional levels revealed by transcriptomic analysis. (B) The transcriptional levels of pMF1.19 and pMF1.20 by qPCR. [file Image_1.tif]

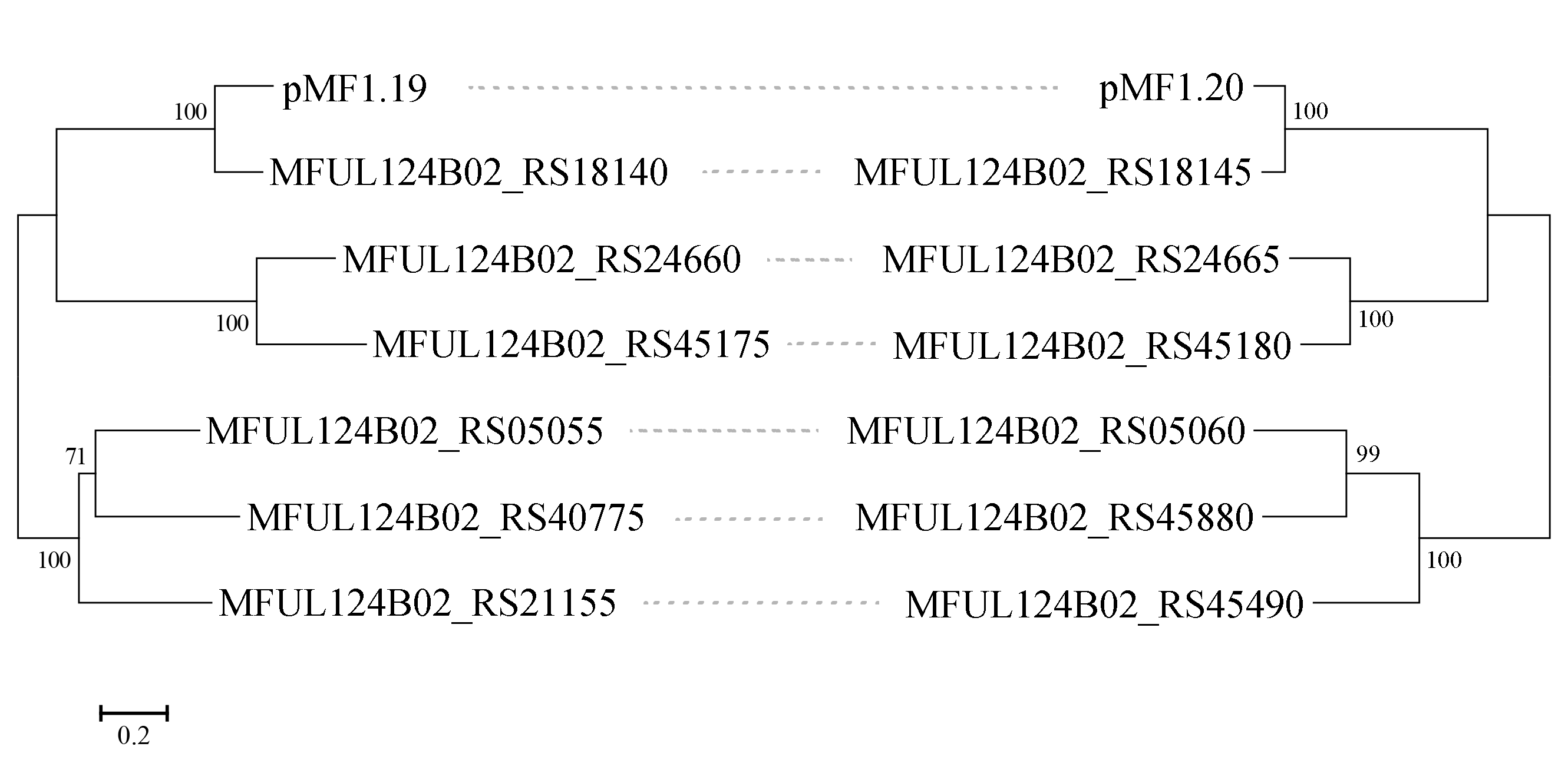

Supplement: Figure S2 — Comparison of the phylogenies of the amino acid sequences of pMF1.19 and pMF1.20 and their homologues in Myxococcus fulvus 124B02. [file Image_2.tif]

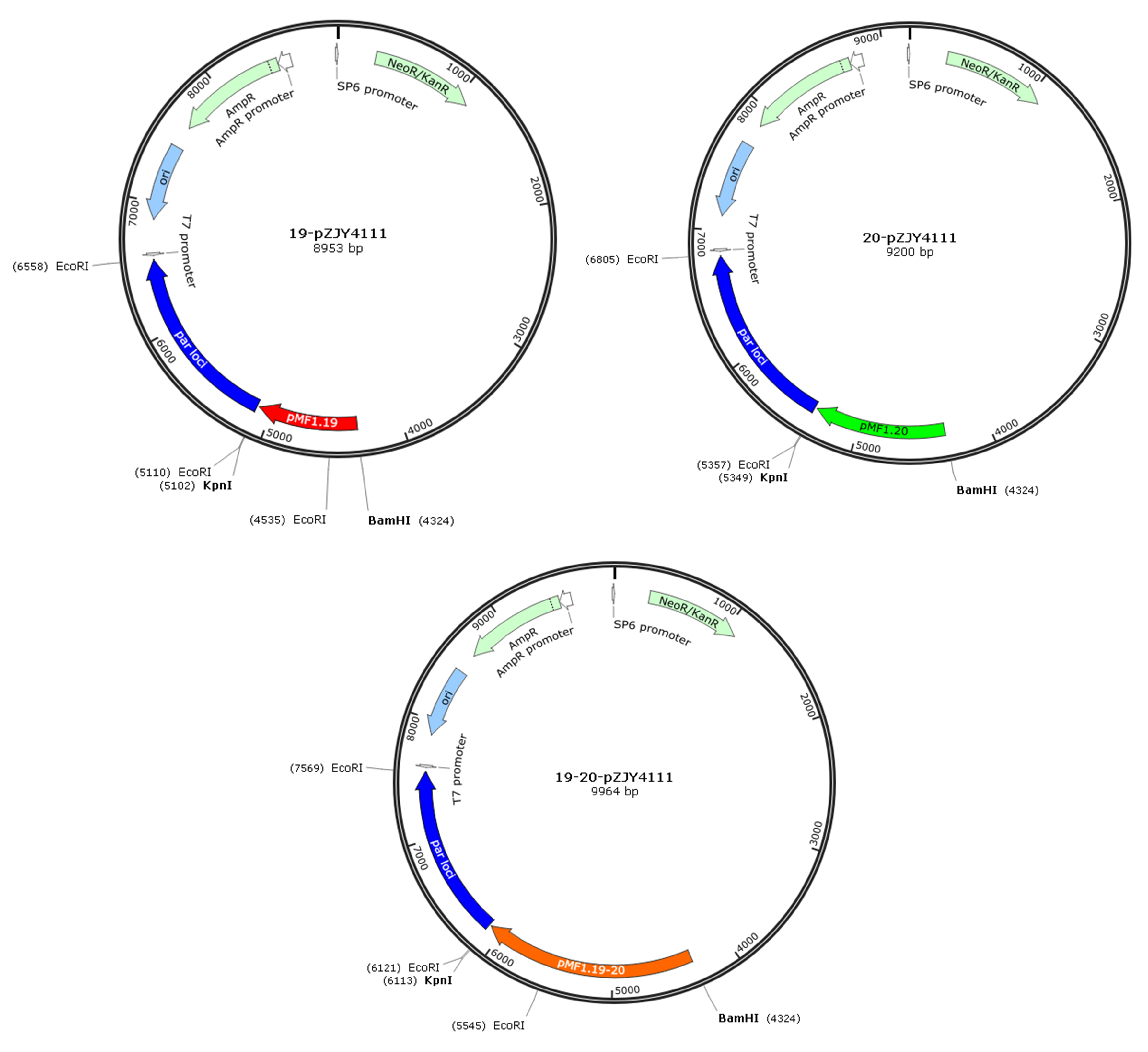

Supplement: Figure S3 — Construction of the 19-pZJY4111, 20-pZJY4111 and 19-20-pZJY4111 plasmids. [file Image_3.tif]

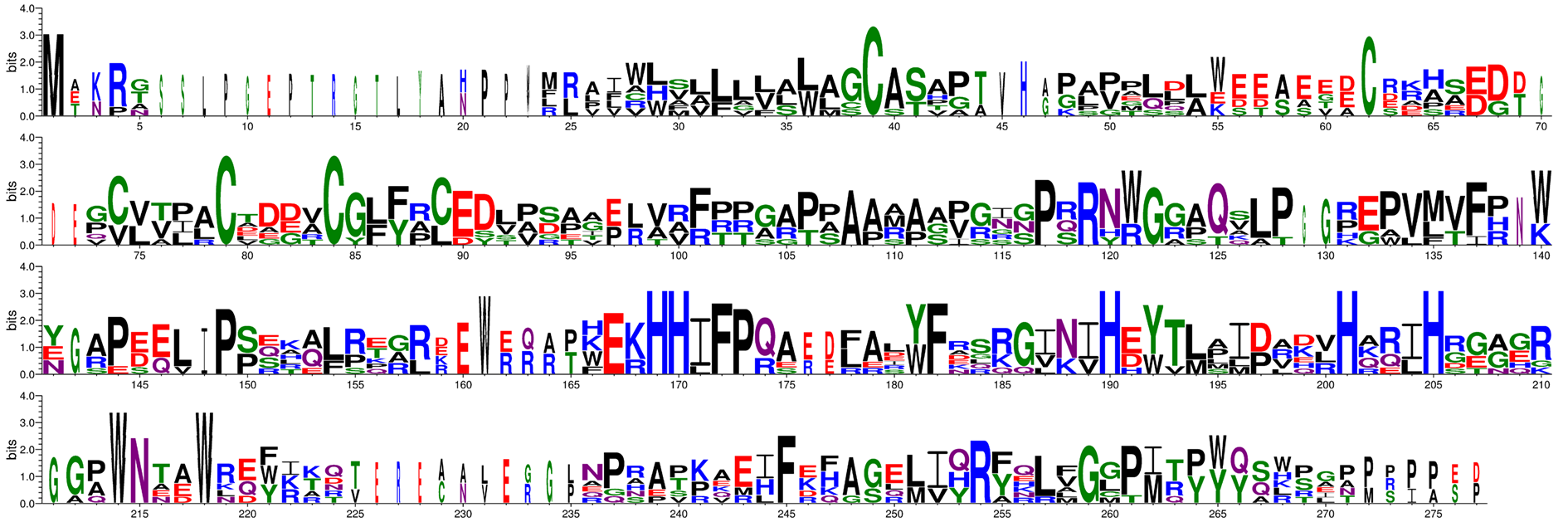

Supplement: Figure S4 — Consensus sequence of pMF1.20 and its homologues in Myxococcus fulvus 124B02. The sequence diversity at each position was assessed by multiple sequence alignment and displayed as a WebLogo. [file Image_4.tif]
