# Supplementary material for: A Post-segregational Killing Mechanism for Maintaining Plasmid PMF1 in Its Myxococcus fulvus Host
Source: Front Cell Infect Microbiol. 2018 Aug 7;8:274. doi: 10.3389/fcimb.2018.00274 (PMC6091211; doi:10.3389/fcimb.2018.00274)
Supplement: Table S1 — Protein Details for pMF1. [file Table_1.docx]

**Table S1. Protein Details for pMF1.**

| **Locus tag** | **Start** | **Stop** | **Strand** | **Protein product** | **Best BLASTp results** | | | | |
| --- | --- | --- | --- | --- | --- | --- | --- | --- | --- |
|  |  |  |  |  | **Sbjct** | **Cover** | **Ident** | **E value** | **Description** |
| pMF1.1 | 803 | 1753 | + | YP_001691200.1 | WP_015349714.1 | 89% | 40% | 4.00E-41 | hypothetical protein [Myxococcus stipitatus] |
| pMF1.2 | 1750 | 2502 | + | YP_001691201.1 | WP_015349715.1 | 96% | 41% | 5.00E-50 | ATP-binding protein [Myxococcus stipitatus] |
| pMF1.3 | 2516 | 2692 | + | YP_001691202.1 | - | - | - | - | - |
| pMF1.4 | 2757 | 4220 | + | YP_001691203.1 | WP_015349717.1 | 94% | 47% | 5.00E-141 | hypothetical protein [Myxococcus stipitatus] |
| pMF1.5 | 4664 | 5530 | + | YP_001691204.1 | - | - | - | - | - |
| pMF1.6 | 6107 | 6571 | + | YP_001691205.1 | - | - | - | - | - |
| pMF1.7 | 6574 | 7332 | + | YP_001691206.1 | WP_015349722.1 | 87% | 43% | 2.00E-38 | hypothetical protein [Myxococcus stipitatus] |
| pMF1.8 | 7329 | 7964 | + | YP_001691207.1 | WP_047855359.1 | 83% | 48% | 3.00E-47 | LysM peptidoglycan-binding domain-containing protein [Archangium gephyra] |
| pMF1.9 | 7961 | 8263 | + | YP_001691208.1 | - | - | - | - | - |
| pMF1.10 | 8275 | 9225 | + | YP_001691209.1 | - | - | - | - | - |
| pMF1.11 | 9264 | 9704 | + | YP_001691210.1 | WP_015349724.1 | 71% | 49% | 6.00E-20 | hypothetical protein [Myxococcus stipitatus] |
| pMF1.12 | 10005 | 11045 | + | YP_001691211.1 | WP_093519318.1 | 56% | 33% | 8.00E-07 | serine/threonine protein kinase [Stigmatella erecta] |
| pMF1.13 | 11250 | 11780 | + | YP_001691212.1 | - | - | - | - | - |
| pMF1.14 | 11777 | 13681 | + | YP_001691213.1 | - | - | - | - | - |
| pMF1.15 | 13678 | 14010 | + | YP_001691214.1 | WP_015349713.1 | 45% | 42% | 2.00E-05 | hypothetical protein [Myxococcus stipitatus] |
| pMF1.16 | 14029 | 14853 | + | YP_001691215.1 | WP_095984790.1 | 70% | 47% | 7.00E-44 | hypothetical protein [Cystobacter fuscus] |
| pMF1.17 | 14974 | 15453 | + | YP_001691216.1 | - | - | - | - | - |
| pMF1.18 | 15443 | 15826 | + | YP_001691217.1 | - | - | - | - | - |
| pMF1.19c | 15823 | 16590 | - | YP_001691218.1 | WP_082165154.1 | 99% | 75% | 2.00E-134 | hypothetical protein [Myxococcus fulvus] |
| pMF1.20c | 16587 | 17309 | - | YP_001691219.1 | WP_082165155.1 | 87% | 88% | 1.00E-127 | DUF2380 domain-containing protein [Myxococcus fulvus] |
| pMF1.21 | 17391 | 17654 | + | YP_001691220.1 | - | - | - | - | - |
| pMF1.22 | 17651 | 18334 | + | YP_001691221.1 | WP_050428907.1 | 95% | 47% | 2.00E-53 | chromosome partitioning protein ParA [Chondromyces crocatus] |
| pMF1.23 | 18336 | 18596 | + | YP_001691222.1 | - | - | - | - | - |
