# Supplementary material for: A Post-segregational Killing Mechanism for Maintaining Plasmid PMF1 in Its Myxococcus fulvus Host
Source: Front Cell Infect Microbiol. 2018 Aug 7;8:274. doi: 10.3389/fcimb.2018.00274 (PMC6091211; doi:10.3389/fcimb.2018.00274)
Supplement: Table S2 — Homologues in Myxococcus fulvus 124B02 of pMF1.19 and pMF1.20. [file Table_2.docx]

**Table S2. Homologues in *Myxococcus fulvus* 124B02 of pMF1.19 and pMF1.20.**

| **Pairs** | **Protein** | **Locus tag** | **BLASTp with 19 (A) or 20 (B)** | | | **Accession** | **Start** | **Stop** | **Strand** | **Description** |
| --- | --- | --- | --- | --- | --- | --- | --- | --- | --- | --- |
|  |  |  |  | **Identities** | **Expect** |  |  |  |  |  |
| 1 | YP_001691218.1 | pMF1.19c | A | - | - | NC_010372.1 | 15823 | 16590 | - | hypothetical protein pMF1.19c (plasmid) |
| 1 | YP_001691219.1 | pMF1.20c | B | - | - | NC_010372.1 | 16587 | 17309 | - | hypothetical protein pMF1.20c (plasmid) |
| 2 | WP_046711024.1 | MFUL124B02_RS05055 | A | 43% | 7.00E-51 | NZ_CP006003.1 | 1187851 | 1188564 | - | hypothetical protein |
| 2 | WP_046711025.1 | MFUL124B02_RS05060 | B | 38% | 3.00E-14 | NZ_CP006003.1 | 1188578 | 1189249 | - | DUF2380 domain-containing protein |
| 3 | WP_082165018.1 | MFUL124B02_RS45175 | A | 34% | 7.00E-32 | NZ_CP006003.1 | 2321566 | 2322312 | - | hypothetical protein |
| 3 | WP_082165019.1 | MFUL124B02_RS45180 | B | 37% | 2.00E-37 | NZ_CP006003.1 | 2322312 | 2323052 | - | DUF2380 domain-containing protein |
| 4 | WP_082165154.1 | MFUL124B02_RS18140 | A | 75% | 2.00E-134 | NZ_CP006003.1 | 4501600 | 4502520 | - | hypothetical protein |
| 4 | WP_082165155.1 | MFUL124B02_RS18145 | B | 88% | 1.00E-127 | NZ_CP006003.1 | 4502376 | 4503059 | - | DUF2380 domain-containing protein |
| 5 | WP_046713620.1 | MFUL124B02_RS21155 | A | 38% | 8.00E-34 | NZ_CP006003.1 | 5334735 | 5335457 | - | hypothetical protein |
| 5 | WP_082165206.1 | MFUL124B02_RS45490 | B | 30% | 3.00E-17 | NZ_CP006003.1 | 5335464 | 5336120 | - | DUF2380 domain-containing protein |
| 6 | WP_046714197.1 | MFUL124B02_RS24660 | A | 34% | 4.00E-32 | NZ_CP006003.1 | 6359462 | 6360211 | - | hypothetical protein |
| 6 | WP_046714198.1 | MFUL124B02_RS24665 | B | 40% | 3.00E-25 | NZ_CP006003.1 | 6360353 | 6360943 | - | DUF2380 domain-containing protein |
| 7 | WP_046716801.1 | MFUL124B02_RS40775 | A | 39% | 3.00E-36 | NZ_CP006003.1 | 10345956 | 10346678 | - | hypothetical protein |
| 7 | WP_082165493.1 | MFUL124B02_RS45880 | B | 33% | 4.00E-16 | NZ_CP006003.1 | 10346685 | 10347356 | - | DUF2380 domain-containing protein |
