# Supplementary material for: A Post-segregational Killing Mechanism for Maintaining Plasmid PMF1 in Its Myxococcus fulvus Host
Source: Front Cell Infect Microbiol. 2018 Aug 7;8:274. doi: 10.3389/fcimb.2018.00274 (PMC6091211; doi:10.3389/fcimb.2018.00274)
Supplement: Table S3 — Strains and plasmid used in this study. [file Table_3.docx]

**Table S3. Strains and plasmid used in this study.**

| **Strain** | **Description** | **Source** |
| --- | --- | --- |
| *M. xanthus* DZ1 | Non-motile, Non-fruiting, dispersed-growing | D. R. Zusman, University of California, Berkeley |
| *E. coli* DH5α | F- endA1 glnV44 thi-1 recA1 relA1 gyrA96 deoR nupG Φ80dlacZΔM15 Δ(lacZYA-argF)U169, hsdR17(rK–mK+), λ– | Life Technologies Inc. |
| *E. coli* BL21 (DE3) | λ(DE3 [lacI lacUV5-T7 gene 1 ind1 sam7 nin5]) [malB+]K-12(λS) | Stratagene Co. |
| **Plasmid** | **Description** | **Source** |
| pMF1 | A cryptic plasmid from *M. fulvus* 124B02, 18.634 kb | Zhao et al., 2008 |
| pZJY4111 | Amp^r^, Km^r^, the stable *E. coli*- *M. xanthus* shuttle plasmid containing *ori* and *par* loci of pMF1 | [Sun et al., 2011](#_ENREF_102) |
| 19-pZJY4111 | Amp^r^, Km^r^, *pMF1.19* insertion in pZJY4111 | This study |
| 20-pZJY4111 | Amp^r^, Km^r^, *pMF1.20* insertion in pZJY4111 | This study |
| 19-20-pZJY4111 | Amp^r^, Km^r^, *pMF1.19-pMF1.20* insertion in pZJY4111 | This study |
| pMAL-c5X | Amp^r^, Tac promoter,MBP Tag,5.7 kb | New England Biolabs |
| 19- pMAL | Amp^r^, *pMF1.19* insertion in pMAL-c5x | This study |
| 20-pMAL | Amp^r^, *pMF1.20* insertion in pMAL-c5x | This study |
| pACYC Duet-1 | Expression vector, the P15A replicon, Cm^r^ | Novagen |
| 19-pACYC | Cm^r^, *pMF1.19* insertion in pACYC-Duet-1 | This study |
| pET28a | Expression vector, Km^r^, N-His-tag, C-His-tag, T7 promotor | Qi Q.S. Shandong |
| 20-pET28a | Km^r^, *pMF1.20* insertion in pET28a | This study |
