# Supplementary material for: A Post-segregational Killing Mechanism for Maintaining Plasmid PMF1 in Its Myxococcus fulvus Host
Source: Front Cell Infect Microbiol. 2018 Aug 7;8:274. doi: 10.3389/fcimb.2018.00274 (PMC6091211; doi:10.3389/fcimb.2018.00274)
Supplement: Table S4 — Primers used in this study. [file Table_4.docx]

**Table S4. Primers used in this study.**

| **Primer name** | **Sequence (5′–3′)*** |
| --- | --- |
| **Primers for the co-transcription PCR detection** | |
| 12-13-up | GCGCTTGATGCTGCCGACGAA |
| 12-13-down | TGGAGCCAGCGGGCTTTCA |
| 13-14-up | ACGCGAAAGCCCTGGCTGACCT |
| 13-14-down | TAGCGGCACTCGGCTCCACATC |
| 14-15-up | GAGTTGACGCGCCGGATTCAAG |
| 14-15-down | ATGTCGACCGCAACAATCCACG |
| 15-16-up | TCGTGCGGTTCGTGTCGCTCAAG |
| 15-16-down | GCTCACCGTCTCCACGTACA |
| 16-17-up | TGGTGGATGGTGGTGAAACGG |
| 16-17-down | CACATACTCCCGGCCTTCCTG |
| 17-18-up | TCCACTCTTACGCACAGGTTCACG |
| 17-18-down | GCATCTCCTCTGGGAGCAACG |
| 18-19-up | TGCGACACGGACTACTGCTCTTC |
| 18-19-down | CGCAACATCCCTGGTCATCGT |
| 19-20-up | ACGTCGCTCGGCTCTAAGG |
| 19-20-down | CATGGTCTTCCACTGGTACGC |
| 20-21-up | TGCGAACCGATGGCCTGGC |
| 20-21-down | GCGTCGGCATCGTGGTCTT |
| 21-22-up | CGAGGATTGAGGAAGAGGTCAGG |
| 21-22-down | ACCACCAGCACGGAAGAGCC |
| 22-23-up | ATCGGATTGCGTGGCAAGAGG |
| 22-23-down | TGAATCTGCACCGTCAGGTAAAGC |
| **Primers for testing plasmid stability** |  |
| BamH I-19-up (4111) | CGGGATCCGCCGCAGACCCCACGAGCGG |
| Kpn I-19-down (4111) | GGGGTACCCTAGCTGCGGCGGATGATG |
| BamH I-20-up (4111) | CGGGATCCGCGTGAGCAGGTTTCCGCGC |
| Kpn I-20-down (4111) | GGGGTACCTCATGGAGGGGGAGGGGGAAGG |
| **Primers for the gene expression in *E. coli*** |  |
| BamH I- 19-up | CGGGATCCGATGACCTACTACGAGGTACAG |
| Hind III-19-down | CCCAAGCTTCTAGCTGCGGCGGATGATGATG |
| Nde I-19-up | GGAATTCCATATGATGACCTACTACGAGGTACAG |
| Nde I- 20-up | GGAATTCCATATGTGTGCGAGCGCACCCACTCCC |
| Hind III-20-down | CCCAAGCTTTCATGGAGGGGGAGGGGGAAG |
